# An Auto-Tuning PI Control System for an Open-Circuit Low-Speed Wind Tunnel Designed for Greenhouse Technology

**DOI:** 10.3390/s150819723

**Published:** 2015-08-12

**Authors:** Karlos Espinoza, Diego L. Valera, José A. Torres, Alejandro López, Francisco D. Molina-Aiz

**Affiliations:** Research Centre BITAL, University of Almería, Ctra. Sacramento s/n, 04120 Almería, Spain; E-Mails: ker154@inlumine.ual.es (K.E.); jtorres@ual.es (J.A.T.); alexlopez@ual.es (A.L.); fmolina@ual.es (F.D.M.-A.)

**Keywords:** low-speed wind tunnel, auto-tuning PI controller, greenhouse agriculture technology

## Abstract

Wind tunnels are a key experimental tool for the analysis of airflow parameters in many fields of application. Despite their great potential impact on agricultural research, few contributions have dealt with the development of automatic control systems for wind tunnels in the field of greenhouse technology. The objective of this paper is to present an automatic control system that provides precision and speed of measurement, as well as efficient data processing in low-speed wind tunnel experiments for greenhouse engineering applications. The system is based on an algorithm that identifies the system model and calculates the optimum PI controller. The validation of the system was performed on a cellulose evaporative cooling pad and on insect-proof screens to assess its response to perturbations. The control system provided an accuracy of <0.06 m·s^−1^ for airflow speed and <0.50 Pa for pressure drop, thus permitting the reproducibility and standardization of the tests. The proposed control system also incorporates a fully-integrated software unit that manages the tests in terms of airflow speed and pressure drop set points.

## 1. Introduction

The surface area of greenhouses worldwide is constantly growing and now exceeds 700,000 ha. In the Mediterranean Basin, greenhouse agriculture exceeds 200,000 ha of cropping surface and bears considerable socioeconomic importance in the region. Greenhouses have also been the real driving force behind the socio-economic and demographic development in the province of Almería (Spain), where the highest concentration of greenhouses in the world is located, with more than 30,000 ha of protected cropping surface [1].

The incorporation of technology has become a constant process aimed at increasing productivity and reducing costs in greenhouse agriculture. A central element in this progress has been the design of efficient greenhouse structures and climate control systems, a process in which the analysis of airflow patterns has become essential [1].

Various field and laboratory techniques have been traditionally used in greenhouse engineering to analyze airflow patterns [2,3,4,5,6], including tracer gas techniques [7], air pressure drop measurements [8], sonic anemometry [9] and simulation models, such as computational fluid dynamics (CFD) [5]. Among them, laboratory techniques and simulation models have become increasingly relevant in recent years due to the cost savings they provide in comparison to field trials.

In this context, wind tunnels have emerged as a useful and cost-effective laboratory technique for the improvement of greenhouse structures [10,11,12], the study of climate control systems [13,14,15,16] and the estimation of the aerodynamic parameters of the experimental models. A number of applications of wind tunnels have been described in the literature on greenhouse engineering, including the determination of drag coefficients for horticultural crops [17], the study of aerodynamic parameters of insect-proof screens [2,18,19,20,21,22] and of greenhouse cooling systems [13,23,24], the assessment of wind erosion on agricultural soils [25,26] and the analysis of insect flight mechanisms [27,28]. However, a recent review of the literature [29] concluded that only 35 out of 75 greenhouse studies performed a verification process using wind tunnel tests or field examinations. In a similar application field, wind tunnels have been successfully employed for the study of artificial substrates in active living walls in Mediterranean buildings [30] and for the analysis of refrigeration systems in buildings [31].

Several control objectives of wind tunnels have been reported in the literature, such as the generation of a turbulent profile, the adjustment of the power spectral density, the reduction of low-frequency pulsations and the suppression of the vibration of the cantilever beam structures that support wind tunnel models. In particular, previous research has simulated a required wind structure by using a wind tunnel with multiple fans controlled independently [32]. In a similar vein, Pan *et al.* [33] proposed a multiple-fan, active-control system to adjust the power spectral density and the integral length in a variety of harmonic wind turbulences. In addition, the results by Nishi *et al.* [34] confirmed a good agreement between target and measured turbulence parameter profiles by using the time-lag modification method. In a more recent study, Wang *et al.* [35] addressed the control of the low-frequency pulsations that affect both the aerodynamics and the acoustic measurements in an open-circuit wind tunnel by adjusting the angles of the collector flaps and drilling an opening in the test section chamber above the nozzle. In another contribution, Li *et al.* [36] proposed a hybrid fuzzy-PID control system for the cantilever beam structure that supports the scale models in order to avoid the drawbacks caused by the low-frequency resonant vibration resulting from the natural frequency induced by the flow.

In this regard, it is important to note that the specific characteristics of the wind tunnel determine the focus of the control methods to be chosen [37]. In this way, previous studies that simulate the boundary layer using low-speed tunnels have focused on the control of turbulence. Similarly, previous research in aeronautics using transonic and supersonic tunnels has focused on the control of airflow speed, since in these tunnels, the airflow is induced at the outlet of a tank of compressed air, which limits the test time and, thus, requires a rapid and precise establishment of the desired airflow speed set point.

In this context, an automatic control system of airflow speed has become a requisite for standardizing the experiments performed in wind tunnels due to the precision and speed of measurement and efficient data collection that it provides.

Since the 1950s, a substantial body of research has been developed on automatic control schemes in supersonic and transonic wind tunnels. As an example of an automatic control scheme, proportional-integral-derivative (PID) control has expanded rapidly due to its effectiveness and simple implementation. In conjunction with neural networks [38], genetic algorithms [39], fuzzy control [40] and predictive control [41], PID control has been used mainly in supersonic [42] and transonic tunnels for controlling pressure and Mach number, as well as for the rapid establishment of airflow speed set points due to the fact that the operation of such wind tunnels is based on compressed air. In a subsonic wind tunnel using a direct current motor, Dinca and Corcau [43] used a three-phase rectifier and a PI control algorithm developed in LabVIEW. Likewise, Xuan *et al.* [44] implemented a proportional feedback control system added to a neuronal network in conjunction with a diffuse controller, resulting in an increase of the robustness of the network.

A major research challenge stems from the fact that control models are characteristically nonlinear, unstable and hard to establish due to the inherent complexity of the wind speed control system. For this reason, there is great interest in applying modeling and control techniques that can be adapted to the diverse experimental models to be tested in the wind tunnel [44]. Moreover, the modeling of the wind tunnel is a requisite for the design process of its controller, since estimation of the control parameters for one operation point does not guarantee stability over the whole spectrum of operation [39]. Hence, a model identification algorithm is still required despite the wide variety of controllers that can be satisfactorily implemented in wind tunnels. Wang *et al.* [37] proposed a decoupled multi-model control scheme for a multi-variable process to be implemented in a wind tunnel. Recently, Zhang *et al.* [45] developed a decoupled multi-variable PID controller that was fitted using a dimensional algorithm for a wind tunnel.

In this context, the Arduino ecosystem, a hardware and software set for the implementation of automatic control and monitoring, has become a powerful tool widely used in an ample range of applications in the fields of education, science, industry and manufacturing due to its cost efficiency and open architecture. The system is based on the Arduino board, which includes an ATmega microcontroller and I/O pins, and the platform has an integrated development environment. These features make the Arduino platform suitable as a substitute for other automatic systems.

The use of automatic systems based on Arduino boards has been tested in the field of agriculture for monitoring the temperature and humidity inside greenhouses [46] and also in subaquatic environments for implementing a control mechanism for an actuator in subaquatic environments in conjunction with the MATLAB environment [47].

Despite the expansion of wind tunnel technology from aeronautics and civil engineering into agricultural research, it is remarkable that few studies have focused on low-velocity wind tunnels in greenhouse agriculture. This paper describes an automatic system to control the airflow speed and pressure drop of a low-speed wind tunnel for greenhouse engineering applications and an integrated software unit to manage the tests. To successfully fulfill its purpose, the proposed solution must meet certain requirements. Notably, the control system must allow the determination of the aerodynamic properties of complex objects, such as corrugated cellulose evaporative cooling pads, insect-proof screens and plants. In other cases, the system must additionally allow the calculation of the mass and energy balances as in the case of corrugated cellulose pads. To control airflow speed or pressure drop in the test section of the wind tunnel, the control algorithm must be able to identify the process model of the wind tunnel and subsequently design a controller by auto-tuning.

Based on these requirements, we begin in Section 2 by describing the proposed hardware and software algorithm to manage and control the low-speed wind tunnel. We then proceed to validate in Section 3 the management and control system on various insect-proof screens and corrugated cellulose pads. Finally, we formulate our conclusions in Section 4.

## 2. Materials and Methods

### 2.1. Wind Tunnel

**Figure 1:**
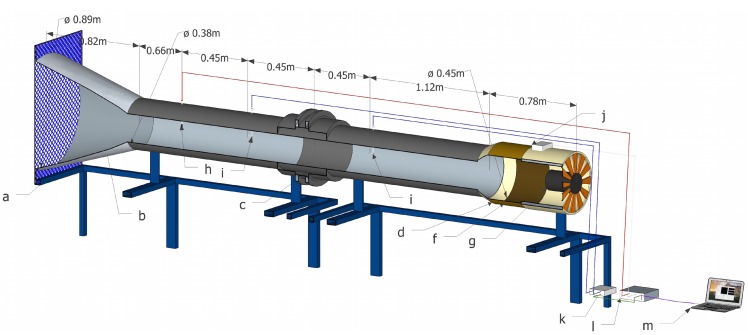
Low-velocity wind tunnel composed of: (**a**) flow conditioner, which uses a grille to control turbulence; (**b**) contraction; (**c**) test section; (**d**) diffuser; (**f**) elastic joint clamp; (**g**) fan; (**h**) hot-film anemometer and temperature probe; (**i**) Pitot tubes; (**j**) frequency inverter; (**k**) pressure transducer; (**l**) electronic interface; (**m**) computer.

The auto-tuning PI control system developed in this study was implemented and validated in a low-speed wind tunnel (Figure 1) designed and developed at the University of Almería [2,17,30,48]. The airflow speed in the wind tunnel could be regulated from 0.10 to 10.00 m/s, corresponding to Reynolds numbers of 2500.00 and 2.50 × 10^5^, respectively, therefore fully within the transient and turbulent regime. The upper airflow speed value corresponds to the threshold above which greenhouse windows are closed in order to avoid damage to the greenhouse structure. The contraction ratio of the wind tunnel was 1:5.32, with a coefficient between the entrance diameter and the length of the contraction section of 0.92 [48]. The equipment allowed insect-proof screens [48], cellulose evaporative cooling pads [13], crops [17] and even the substrates used in active living walls [30] to be tested.

The pressure drop was recorded by two Pitot tubes of 4.00 mm in diameter (Airflow Developments Ltd, Buckinghamshire, UK) connected to a differential pressure transducer SI727 (Special Instruments, Norlingen, Germany) with an operational range of 0 to 200.00 Pa, a precision of ±0.25 % FS and an output signal of 0 to 10.00 V. Airflow speed was measured by a hot-wire anemometer EE70-VT32C5 (Elektronik, Engerwitzdort, Austria), with a measurement range of 0 to 10.00 m/s, a precision of ±0.20 m/s, a response time of <1.50 s at 10.00 m/s and a signal of 0 to 10.00 V. The flow in the wind tunnel was induced by an axial fan, Model HCT-45-2T-3/AL (Sodeca S.A., Sant Quirze of Besora, Spain), controlled by an alternating-current frequency changer, Micromaster 420 (Siemens Energy & Automation Inc., Alpharetta, GA, USA), with linear control *V/f* over the range of input signals from 0 to 10.00 V and of output signals from 0 to 50.00 Hz. For the tests of the cellulose evaporative pads, a submersible centrifugal pump was used, Model Amazon-LVM105 (Wind & Son Ltd., Herefordshire, UK) featuring 12.00 V, 4.30 A and a maximum flow of 18.00 L·min^−1^. The flow of water was measured using a rotameter with a measurement range of 3.00 to 22.00 L·min^−1^ and a measurement error of ±4.00 %.

### 2.2. Control Circuit and Data Management

The previously-used hardware of the control system was designed to work with a software unit [14] that did not allow an automatic control of the airflow speed and pressure drop in the wind tunnel. The system was designed to work at a fixed measuring speed of 0.33 Hz, thus resulting in a limited test performance. The previous version of the hardware and software unit was based on obsolete electronic components, which hampered the implementation of new control techniques. The maximum test process speed was constrained to the limited process speed of 5 MIPS (million instructions per second) of the PIC16F876 microprocessor. In addition, the tests with this system were performed with voltage set points, which did not allow for reproducibility. For all of these reasons, it was decided to replace the electronic circuit as a means to improve the test management software, to implement an automatic control system and to keep the system open for future improvements.

To this end, an electronic circuit was designed to acquire data from the sensors as previously described (Section 2.1), as well as a control system for the frequency inverter through which the fan speed is controlled. Arduino Uno R3 (Arduino SRL, Turin, Italy) was used for this purpose. The microcontroller on the Arduino board is based on the ATmega328 with a processing speed of 20 MIPS. The board features fourteen digital input/output pins (of which, six can be used as pulse-width modulation (PWM) outputs), six analog inputs, a 16 ceramic resonator, a USB connection and is powered through an AC-to-DC adapter.

**Figure 2:**
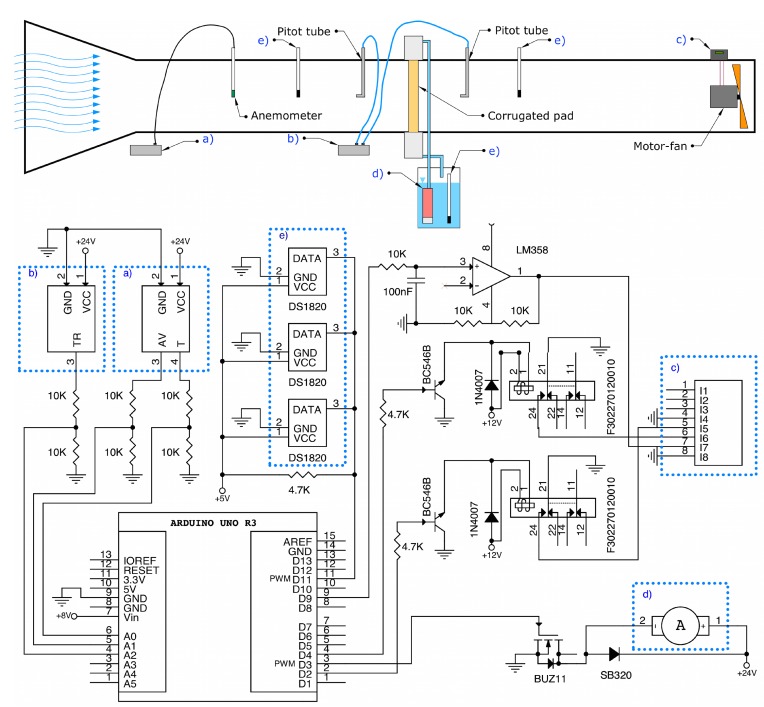
Electronic interface. (**a**) Signal conditioner of the anemometer; (**b**) Pitot tubes and differential pressure transducer; (**c**) frequency inverter; (**d**) pump; (**e**) thermometers.

With a digital signal from the Arduino to the transistors BC546 connected to two relays (Figure 2) supplied with 12.00V, the motor can be switched on and off and its rotation direction changed by using the frequency inverter.

The Arduino board generates an output signal in the range of 0 to 5.00 V. Through its 10-bit A/D converter, a resolution of 4.90 mV can be obtained, and the output signal is then delivered to the frequency inverter. Arduino’s Timer1 Library was used to generate an analog output signal, assisted by a low pass filter and an operational amplifier LM358N supplied at 12.00 V. By this means, we obtained an analog signal of 0 to 10.00 V in the frequency inverter.

The signal of the sensors (0 to 10.00 V) was adjusted by implementing a voltage splitter, so that the Arduino board could take readings of the sensors in the range 0 to 5.00 V.

To allow for future modifications of the circuit, a proportional control of the centrifugal pump for the tests of the cellulose evaporative cooling pads was implemented by using a MOSFET BUZ11 transistor supplying the motor at 12.00 V. The pump was controlled using pulse-width modulation (PWM) signal from the Arduino board. Finally, a network of 1-Wire DS1820 temperature sensors was also added to the system.

### 2.3. Development of the Software

**Figure 3:**
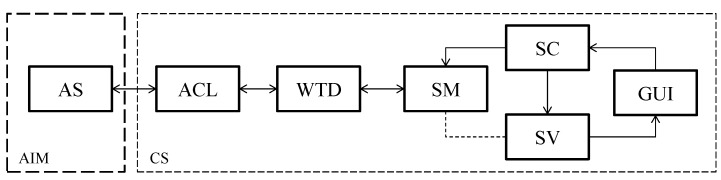
Representation of the developed software algorithm.

The developed software algorithm for the management and control of the wind tunnel consisted of two parts (Figure 3). Firstly, the Arduino information manager (AIM), the algorithm for the management of information on the Arduino board, developed in C/C++. Secondly, the computer software (CS) developed with MATLAB object-oriented programming for the control, management and storage of data.

On the one hand, the Arduino information manager (AIM) consisted of a switch-case block for reading to or writing data from the pins if requested from the computer and also identifies the Arduino board via the USB port to the computer. This software section manages the information coming from the Arduino software (AS) located on the Arduino board.

On the other hand, the CS comprised the following objects: (1) The Arduino communication library (ACL) developed for the communication of the software with the Arduino board; (2) The wind tunnel driver (WTD), a specific driver for the wind tunnel that keeps the methods for requesting the identifier of the Arduino and the readings from the sensors, sends output to the frequency changer and controls the pump; (3) the software model (SM), an object that contains the methods for the system identification, auto-tuning PI algorithm, the implementation of the controller and the algorithm for tests management; (4) the software controller (SC), developed to manage the orders from the GUI, update the variables and run the SM methods; (5) the software view (SV), an object that updates the GUI according to the state of the SM variables, and finally; (6) The graphic user interface (GUI) that contains the user’s control objects.

**Figure 4:**
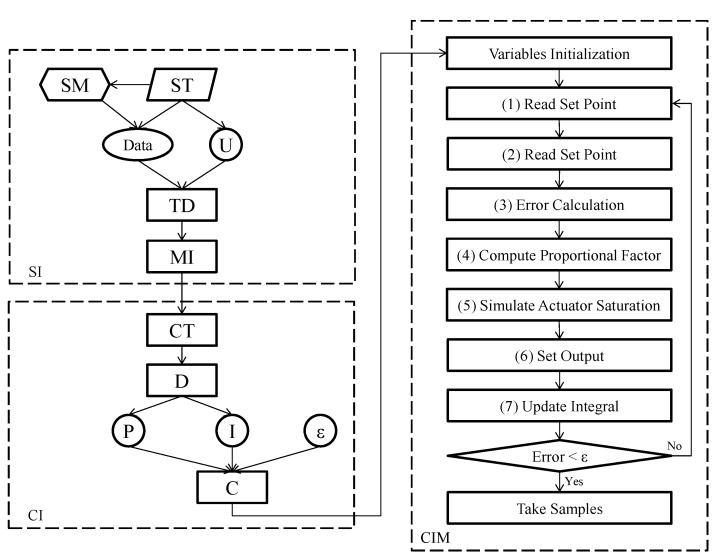
Software model.

The previously-described objects on the CS run four main processes (Figure 4): (1) the system identification (SI) function for identifying the system wind tunnel; (2) the controller identification (CI) function for identifying the controller; (3) the controller implementation (CIM) function for implementing the controland; (4) the test management (TM) that calculates the sequence of set points and manages and stores the test data.

#### 2.3.1. System Identification

For the design of the controller, the algorithm was designed to generate a reaction curve for the system by measuring airflow speed and pressure drop samples at a frequency of 1/ST, where *ST* is the sampling time determined by the user, with a step signal *U* in volts for the frequency inverter (Figure 4). Seventy samples were determined empirically. The first 10 samples were intended to pre-define the system’s initial conditions in absence of air movement; the next 50 samples were aimed at recording the step response; and the last 10 samples were intended to record the maximum mean amplitude $A_{max}$. The maximum amplitude of this signal was limited to 5.00 and was determined by the user or by a tracking algorithm; moreover, $A_{max}$ was determined by the measurement values of the sensors (transducer and anemometer).

A reaction curve for pressure drop and airflow speed (*Data*) using airflow speed samples (*SM*) and *ST* was generated by the system. Changes in the experimental model (*i.e.*, insect-proof screens, cellulose evaporative cooling pads, crops, *etc*.) resulted in variations of the energy required to generate the speed and pressure drop conditions. The reaction curve in the time domain *TD* was then used in the model implementation (*MI*) to calculate a Single-input single-output system (SISO) model with a single pole for both airflow speed and pressure drop.

#### 2.3.2. Controller Identification

The controller identification process CI used the controller tuning (CT) function based on the *pidtune* algorithm by MATLAB, which auto-designs a parallel PI controller for both of the previously-identified models, *i.e.*, the airflow speed model and pressure drop model. The algorithm optimized the closed-loop stability, with adequate performance and robustness. By default, the algorithms choosed, a crossover frequency (loop bandwidth) based on the plant dynamics, and designed, the controller for a target phase margin of 60.00 [49], were used. Since each controller was designed for a system in continuous time, the controller was approximated in discrete time for subsequent digital implementation, to obtain the discrete proportional (*P*) and integral (*I*) values. The permissible error (*ϵ*) was defined by the user.

#### 2.3.3. Controller Implementation

Once airflow speed and drop pressure models and controllers were calculated, the variable (*i.e*., airflow speed or pressure drop) to control was selected by the user. This variable was then used by the CIM process for its control scheme and in the following test management process. The implementation of the controller CIM (Figure 4) consisted of an algorithm loop, which consecutively executed the following stages: (1) reading of the set point indicated by the user (airflow speed or pressure drop); (2) reading of the process variable (airflow speed or pressure drop); (3) error calculation; (4) computation of the temporary output using the current proportional value and the earlier integral value; (5) simulation of the actuator saturation to avoid overshoots in case of an excessive control signal; (6) output setup and; (7) update of the integral variable using numerical integration by the backward Euler method. This process was executed until the error fell below the permissible error (*ϵ*), at which point test samples were taken.

**Figure 5:**
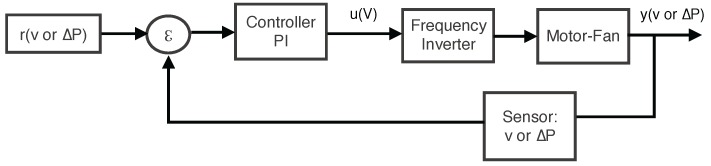
Software model.

The error (*ϵ*) was calculated for each set point (*r*) and sensor reading (Figure 5) and was multiplied by the proportional factor of the controller and integrated to generate the control signal (*u*). This control signal was then sent to the frequency inverter. In turn, the velocity of the fan motor was regulated together with the control V/f, thus producing the output value (*y*). The process was then repeated with a new reading of the sensors.

#### 2.3.4. Test Management

The GUI was used to manage the tests that generated the curves for pressure drop as a function of airflow speed. The developed software unit allowed these curves to be produced based on predefined speed values and then recorded the associated pressure drop, and *vice versa*, by predefining the pressure drop and then measuring the associated velocities.

The curve for pressure drop *vs*. airflow speed (PDAS) could be generated over a range $\left[a_{1},a_{k}\right]$ with a series of set points $S_{k}$ (either airflow speed or pressure drop) heterogeneously distributed (Equation (1)) by *h*. This series of set points is described by:

(1) $$S_{k}=\left(a_{1}+h\right)+\left(a_{2}+h\right)+\cdots +\left(a_{k-1}+h\right)+a_{k}$$

$h=\left(a_{k}-a_{1}\right)/\beta$, where *β* is the number of set points in the series and β∈N.

To generate a PDAS curve with heterogeneously-distributed set points, the values of the series $S_{k}$ were manually assigned:

(2) $$S_{k}=\left(a_{1}+h_{1}\right)+\left(a_{2}+h_{2}\right)+\cdots +\left(a_{k-1}+h_{k-1}\right)+a_{k}$$

where a set point $a\in [\epsilon ,A_{max}]$ in both types of tests and $A_{max}$ is the mean amplitude of the last ten of the 70 stored samples in the reaction curve for the calculation of the controller. The maximum amplitude was reached in the last ten samples; the algorithm then calculated the mean of the last ten samples to obtain a representative value of the maximum amplitude reached for the step response process. Seventy samples were considered empirically as a safe number of recorded observations of the system response to a fan volt step signal. *ϵ* was defined as the minimum permissible error for airflow speed or pressure according to the type of test being executed.

In addition, the algorithm included options for the execution sequences of the set points: increasing (Equation (3)), decreasing (Equation (4)), increasing-to-decreasing with coincident set points (Equation (5)) and increasing-to-decreasing with non-coincident set points (Equation (6)).

(3) $$\begin{array}{ccc} k_{n}=n & k_{n}<k_{n+1} & n\in \left[1,N\right] \end{array}$$

(4) $$\begin{array}{ccc} k_{n}=N+1-n & k_{n}>k_{n+1} & n\in \left[1,N\right] \end{array}$$

(5) $$k_{n}=\left\{\begin{array}{ccc} n & k_{n}<k_{n+1} & n\in \left[1,N\right]\\ N+1-n & k_{n}>k_{n+1} & n\in \left[1,N\right] \end{array}\right.$$

(6) $$k_{n}=\left\{\begin{array}{ccc} & N=2i+1 & i\in N\\ 2n-1 & k_{n}<k_{n+1} & n\in [1,(N+1)/2]\\ N+1-2n & k_{n}>k_{n+1} & n\in [1,(N-1)/2]\\ & N=2i & i\in N\\ 2n-1 & k_{n}<k_{n+1} & n\in [1,N/2]\\ N+2-2n & k_{n}>k_{n+1} & n\in [1,N/2] \end{array}\right.$$

The later set of tests (Equation (6)) was designed mainly to avoid hysteresis errors in the pressure transducer. Moreover, it was possible to assign the number of samples to be stored per set point. In addition, all of the test parameters can be stored in a MATLAB file; similarly, the samples can be stored in a Microsoft Excel file. The mean of the samples per set point was shown graphically in real time (airflow speed *vs*. pressure drop) and in a table. To develop a heterogeneously-distributed set points test; set points could be indicated directly on a table by the user. The number of samples per each set point to be recorded was entered by the user, and airflow speed, pressure and temperature samples were stored in a table directly accessible by the user. The management of the controller and the test is designed to require the least-possible intervention by the user and a fast and precise development of the aerodynamic analysis.

### 2.4. Validation of the Control System

The automatic control system and management of tests were validated by testing two experimental models: a corrugated cellulose evaporative cooling pad and six insect-proof meshes. To validate the controller of airflow speed and pressure drop, a 100.00 mm-thick corrugated cellulose pad set at 45.00° to 45.00° was used. A detailed description of the panel tested is given in Franco *et al.* [13]. For the geometric characterization of the insect-proof meshes (Table 1), a methodology previously developed by the University of Almería was employed [16,50,51].

**Table 1:** Geometric characteristics of the insect-proof screens studied. $D_{r}$, thread density (threads·cm^−2^); *φ*, porosity (%).

Screen	$D_{r}$	*φ*
A	13.10×30.50	0.39±0.01
B	9.90×19.70	0.34±0.01
C, D	9.20×20.70	0.38±0.01
E	10.10×20.00	0.38±0.01
F	9.60×20.30	0.36±0.01

For the first experimental model, four evaluations were performed to validate the system with the corrugated cellulose pad. In Evaluation 1 (Table 2), thirty samples per set point were taken for twelve homogeneously-distributed set points, over an airflow speed range of 0.30 to 3.00 m/s according to the sequences expressed in Equations (3)–(6). The majority of studies [2,17,23,52,53] focus on this airflow speed interval (0.30 to 3.00 m/s), since the negative effect on ventilation, e.g., insect-proof screens, basically occurs at low wind speeds.

**Table 2:** Summary of tests.

	Evaluation	Set Point			Samples/Set	Sequence (Equation)
		No.	Minimum (m·s^−1^)	Maximum (m·s^−1^)		
Pad	1	12	0.30	3.00	30	3, 4, 5 and 6
45°-45°	2	12	0.30	3.00	30	3, 4, 5 and 6
100	3	12	0.30	3.00	30	6
	4	12	0.30	3.00	30	6
Screen A	1	12	0.30	3.00	30	6
Screen B	1	12	0.30	3.00	30	6
Screen C	1	12	0.30	3.00	30	6
Screen D	1	12	0.30	3.00	30	6
Screen E	1	12	0.30	3.00	30	6
Screen F	1	12	0.30	3.00	30	6

Evaluation 2 was designed to assess the control system when it was subjected to a perturbation during a test. Based on the fact that a water film in the corrugated cellulose pad causes a pressure drop change [24], Evaluation 2 was performed in the same way as Evaluation 1, but also causing a perturbation to the system during the control process by applying a flow of water of 7.50 L/min to the corrugated evaporative cellulose pad.

To observe the effect of a continuous application of 7.50 L/min of water to the corrugated cellulose pad during a test, Evaluation 3 was performed by applying this continuous flow and taking thirty samples per set point for twelve homogeneously-distributed set points over the airflow speed range of 0.30 to 3.00 m/s for the sequence expressed by Equation (6).

Evaluation 4 was performed to assess the controller designed as a function of the pressure drop and was executed by taking thirty samples per set point for twelve homogeneously-distributed set points over the pressure range of 5.00 to 50.00 Pa for the sequence expressed in Equation (6).

The second experimental model used to validate the system consisted of a variety of insect-proof screens. The geometric characteristics of each insect-proof screen generated a different reaction in the wind tunnel system. The porous medium of the screens obstructed the passage of airflow, thus requiring more energy to be applied to the fan to produce a given airflow speed or drop pressure. Experiments were performed on six insect-proof screens in homogeneous tests for twelve homogeneous set points and thirty samples per set point; and in increasing-to-decreasing sequence for non-coincident set points over the range 0.30 to 2.90 m/s. A controller was calculated for each mesh, and the PDAS curve was determined using the sequence expressed in Equation (6).

As a result, the four evaluations with the evaporative cooling pad and the evaluation with the six insect-proof screens (each screen with three samples) allowed the performance of the new control system to be determined. All tests were performed at a frequency of 1.00 Hz, which is a safety frequency considering the maximum measured airflow speed and the anemometer time response.

## 3. Results and Discussion

The aforementioned methodology permitted an in-depth study of a novel auto-tuning PI automatic control system for an open-circuit low-speed wind tunnel with application in greenhouse technology.

### 3.1. Graphical User Interface

**Figure 6:**
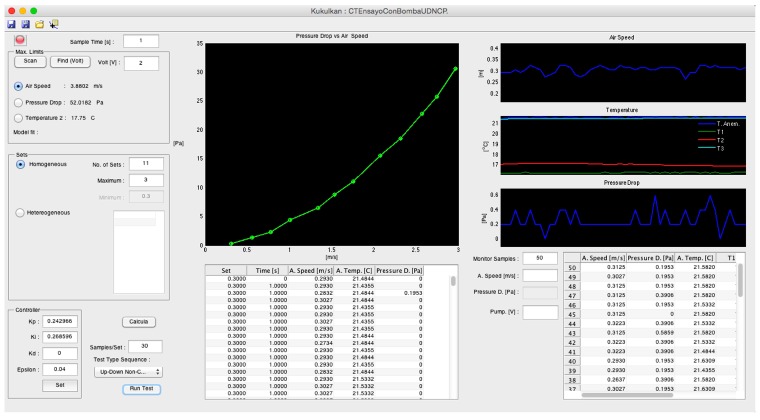
Graphical user interface. Identification of the controller (**upper left**), management of the tests (**left** and **center**) and monitoring in real time (**right**).

The design of the GUI was divided into three sections (see Figure 6): (1) identification of the controller; (2) management of the tests in the sequence; and (3) management of manual tests and real-time monitoring. The maximum amplitude of the reaction curve to adjust the controller could be managed manually in the section assigned to the identification of the controller; similarly, the maximum amplitude could be determined by means of a tracking process. In addition, the parameters PI and permissible error *ϵ* of the control could be manually readjusted. The section dedicated to the management of the tests used homogeneously- or heterogeneously-distributed set points in a range. In addition, this section provided the indication of the type of sequence for executing the set points and the number of samples per set point. The central part of the GUI displayed the mean readings in real time (*i.e.*, airflow speed and pressure drop) for each set point of the sensors over the duration of the test. Finally, the monitoring section in real time displayed a set of three graphs indicating airflow speed, air temperature in the anemometer and pressure drop measured by a differential pressure transducer, as well as a table with the real-time numerical data.

### 3.2. Automatic Identified Models and Auto-Tuned Controllers

An algorithm was developed to obtain the system model and controller for both variables: airflow speed and pressure drop. To obtain the reaction curve, the first step was to determine the amplitude (in volts) of a step signal to the fan. This amplitude was determined manually by indicating the desired maximum voltage that the system was to be assessed for, or by using a maximum-amplitude search algorithm conditioned by an upper measurement limit of 10.00 m/s and 200.00 Pa for the anemometer and pressure transducer, respectively. Once the maximum amplitude was determined, the initial condition was established as a static flow; after this, the reaction curve was generated by recording the time series of the airflow speed and the pressure drop. The parameters of the models were estimated using an algorithm based on regularized estimates with a time constant (pole).

By calculating the model using the *pidtune* algorithm by MATLAB, the controller was auto-tuned to optimize the stability of the closed-loop system, the response time and the margin of stability. The parameters of the proportional and integral controller and the permissible error could be manually modified afterwards, hence providing a reference to the user.

To validate the operation of the auto-tuning algorithm of the model and controller, we obtained eight models and controllers as a result of the two tests performed with the corrugated cellulose pad and the six tests performed for the insect-proof screens.

#### 3.2.1. Cellulose Evaporative Cooling Pad

**Figure 7:**
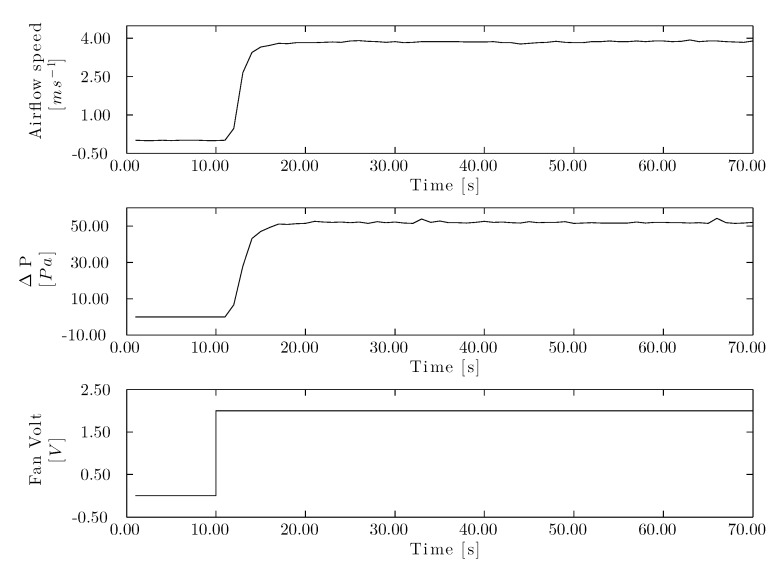
Airflow speed and drop pressure step response in the cellulose evaporative cooling pad. Reaction curve for airflow speed (**top**), reaction curve for pressure drop (**center**) and fan voltage step signal (**bottom**).

The experimental corrugated cellulose pad was subjected to low speeds of 0 to 3.00 *m/s*. To generate a reaction curve for airflow speed and pressure drop (Figure 7), an amplitude of 2.00 V was used in the step signal.

**Figure 8:**
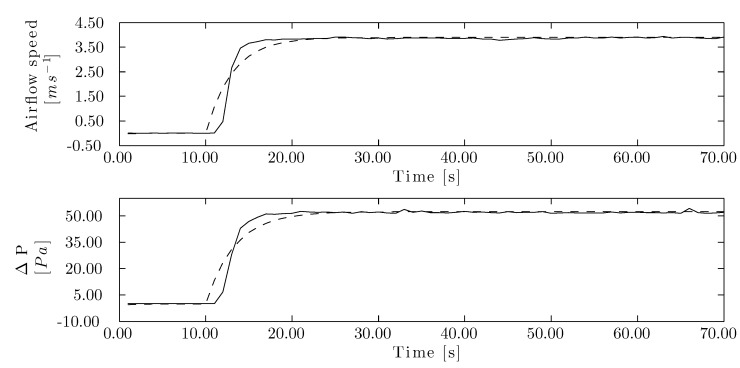
Process reaction curve for the cellulose evaporative cooling pad. Airflow speed reaction curve (**top**) and pressure drop reaction curve (**bottom**). (–) Measured data, (- -) modeled data.

It is noteworthy that the maximum amplitude of each of these curves varied substantially: 3.90 m/s and 54.10 Pa, for the airflow speed and pressure drop, respectively. After the reaction curves were stored, the model was calculated for each curve (Figure 8) by the MI algorithm. The resulting transfer function are expressed in Equations (7) and (8) for airflow speed and pressure drop, respectively. The resulting fit was 82.90 % for the airflow speed and 84.00 % for the pressure drop. Each model was calculated with one pole, thus sacrificing the flexibility in order to increase model precision.

(7) $$G\left(s\right)=\frac{1.95}{1+3.07\cdot s}$$

(8) $$G\left(s\right)=\frac{26.17}{1+3.37\cdot s}$$

**Figure 9:**
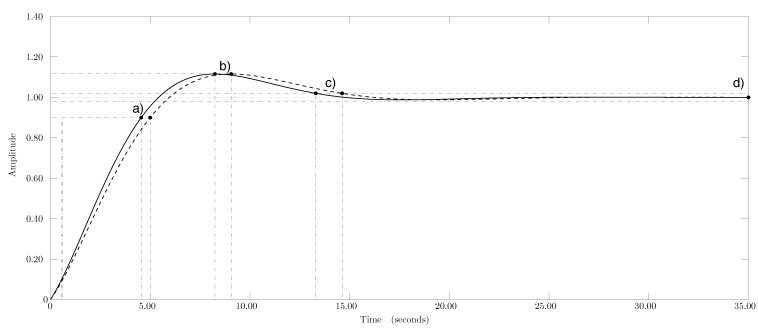
Closed loop system step response of corrugated cellulose evaporative pad. (–) Airflow speed and (- -) drop pressure controller step response. (**a**) Rise time, (**b**) peak response; (**c**) settling time; and (**d**) steady state.

The designed airflow speed controller delivered a proportional factor of 0.24 and an integral one of 0.27, while these values were 0.02 and 0.02, respectively, for the pressure drop controller. The closed-loop system of the wind tunnel was simulated with a step signal (Figure 9) to assess the design of the control system. The rise time was shorter for airflow speed control (3.99 s) than for the pressure drop control (4.38 s). Overshot was 11.20 % for both control processes. The speed controller reached a settling time of 2.00 % in 13.30 s, while it took 14.60 s for the pressure drop controller. The controllers were designed for a phase margin of 60.00◦. The airflow speed controller for the corrugated cellulose pad was adjusted to a crossover frequency of 0.37 *rad/s* and 0.33 *rad/s* for the airflow speed and pressure drop controllers, respectively.

#### 3.2.2. Insect-Proof Screens

For the insect-proof screens, the SI and CI algorithms follow the same process as described in Section 3.2.1. The control system was assessed by means of six insect-proof screens to determine the precision of the control system under a variety of conditions. The models and controllers for each insect-proof screen can be extracted from Table 3. The identification algorithm obtained a fit exceeding 86.00 for all of the insect-proof screens. The models showed slight variations among them, which therefore resulted in variations in the controllers.

**Table 3:** Models and controllers for each anti-insect mesh. The gain Km and the first time constant Tm of the model, proportional gain Kp and integral gain Ki of the controller.

		Transfer function		Model Fit	Controller	
Screen	Repetition	Km	Tm	%	Kp	Ki
A	1	1.57	5.84	87.24	0.30	0.17
	2	1.60	6.03	87.50	0.30	0.17
	3	1.61	6.07	87.50	0.29	0.16
B	1	1.50	6.08	87.97	0.31	0.18
	2	1.45	6.32	88.05	0.33	0.18
	3	1.47	6.03	88.05	0.32	0.18
C	1	1.69	5.51	86.54	0.28	0.17
	2	1.69	5.77	88.84	0.28	0.16
	3	1.56	6.37	88.84	0.30	0.16
D	1	1.60	6.13	88.25	0.30	0.16
	2	1.65	5.78	86.63	0.29	0.17
	3	1.64	5.53	86.63	0.29	0.18
E	1	1.65	5.49	86.38	0.29	0.18
	2	1.61	5.63	87.34	0.29	0.18
	3	1.65	5.71	87.34	0.29	0.17
F	1	1.69	5.33	85.52	0.28	0.18
	2	1.72	5.45	87.28	0.28	0.17
	3	1.66	5.70	87.28	0.29	0.17

### 3.3. Controller Behavior for the Corrugated Cellulose Evaporative Cooling Pad

Airflow speed and drop pressure controllers for the corrugated cellulose evaporative pad were evaluated firstly with just one airflow speed set point, then as described in Table 2.

#### 3.3.1. Airflow Speed Controller Evaluation

##### Test with one set point

**Figure 10:**
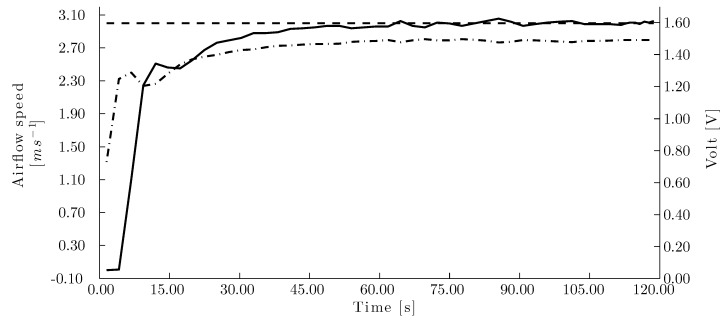
Single velocity set point using the manual control for the corrugated cellulose pad. (- -) Airflow set point, (–) measured airflow speed and (- · -) voltage signal to the fan.

The airflow speed controller test with one set point (3.00 m/s) revealed a settling time of 67.00 s (Figure 10) and a mean deviation of the set point of 0.06 m/s. The test showed the high degree of accuracy when settling the set point despite that the system is subjected to noise due to turbulence.

##### Evaluation 1

**Figure 11:**
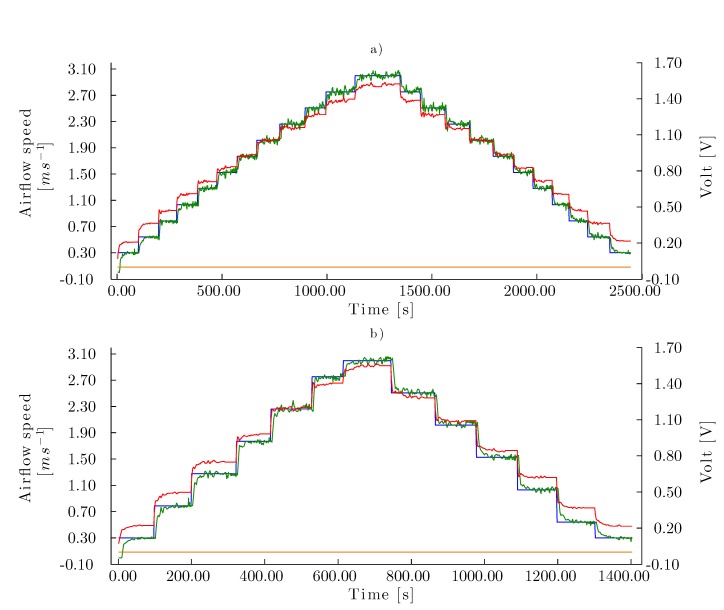
Control curves for Test 1 with the corrugated cellulose evaporative pad. Airflow speed set point series are homogeneously distributed (Equation (1)) in (**a**) increasing-to-decreasing sequence with coincident set points (Equation (5)) and (**b**) increasing-to-decreasing sequence with non-coincident set points (Equation (6)). (–) Airflow speed set points (m·s^−1^), (–) airflow speed measure (m·s^−1^), (–) control signal to fan (V), (–) signal to pump (V).

To assess the performance of the airflow speed controller for the corrugated cellulose pad, airflow speed set points for all of the sequences described in Section 2.3.4 were tested and airflow samples and the control voltage were accordingly recorded. Representative tests are shown in Figure 11. The test time for the corrugated cellulose pad was longer for the increasing-to-decreasing sequence (Equation (5)) since the test sets twice the set point series were compared to the increasing (Equation (3)) and decreasing (Equation (4)) sequences. Nevertheless, the test required a longer testing period, even when the test with the increasing-to-decreasing sequence with non-coincident set points (Equation (6)) had the same number of set points as the increasing (Equation (3)) and decreasing (Equation (4)) sequences. The amplitude between the set points affected the test time, since the amplitude in the increasing-to-decreasing sequence with non-coincident set points (Equation (6)) was twice that of the other sequences.

##### Evaluation 2

**Figure 12:**
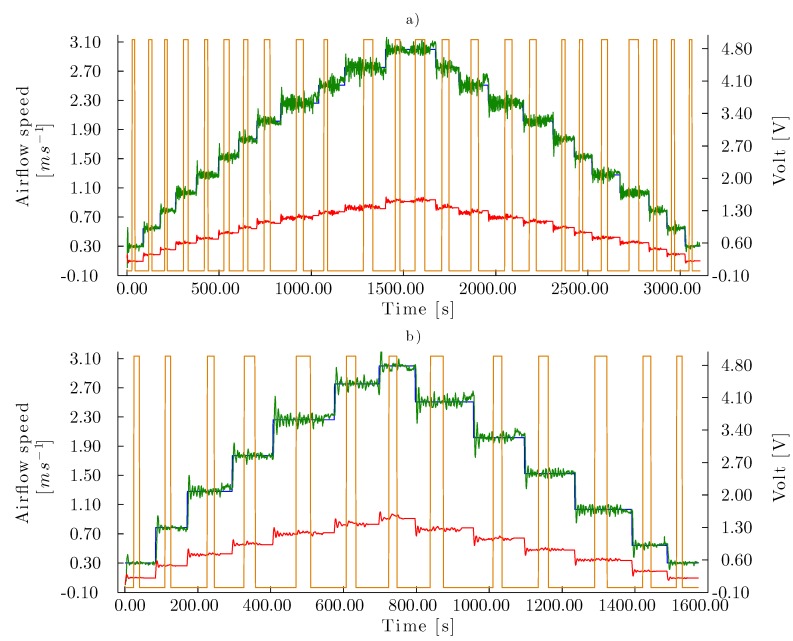
Control curves for Test 2 with the corrugated cellulose evaporative pad. Airflow speed set points series are homogeneously distributed (Equation (1)) in (**a**) increasing-to-decreasing sequence with coincident set points (Equation (5)) and (**b**) increasing-to-decreasing sequence with non-coincident set points (Equation (6)). (–) Airflow speed set points (m·s^−1^), (–) airflow speed measure (m·s^−1^), (–) control signal to fan (V), (–) signal to pump (perturbation)(V).

To control the airflow speed when the aerodynamic parameters of the experimental model changed during a test, it was necessary that the automatic control system responded to these perturbations to maintain the stability of the system. Using the same sequences as in Evaluation 1, the robustness of the control system was analyzed in Evaluation 2 as the injection of water flow (7.50 L/min) onto the corrugated cellulose evaporative cooling pad was eased (Figure 12). The execution time of the test subjected to perturbations was approximately 5.00 min longer than that of the tests from Evaluation 1 in absence of perturbations. Despite maintaining the set point in the permissible error range, there was significant noise during the control.

##### Evaluation 3

**Figure 13:**
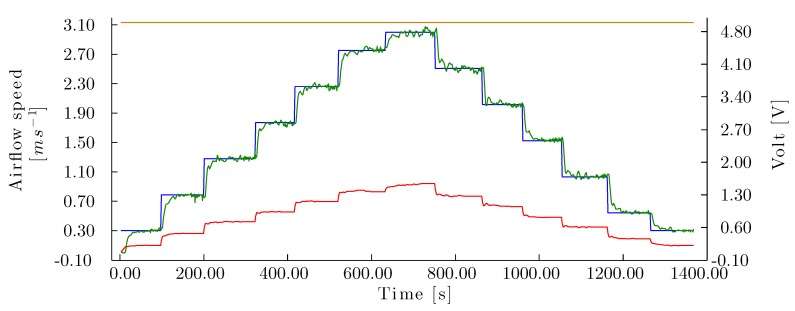
Control curve for Test 3 with the corrugated cellulose pad. (–) Airflow speed set point (m·s^−1^), (–) measured airflow speed (m·s^−1^), (–) signal to fan (V), (–) signal to pump (V).

Evaluation 3 was performed to determine the behavior of the system subjected to a constant perturbation, for which a flow of 7.50 m/s was applied. The flow decreased the corrugated evaporative cooling pad porosity, thus causing a greater pressure drop and changes in the conditions of the system to be controlled (Figure 13). The controller was capable of executing the whole increasing-to-decreasing sequence with non-coincident set points (Equation 6) similarly to when the panel was not subjected to any perturbation (evaluation 2). The test was performed in 1368.51 s.

Using the previous system and the same corrugated evaporative cooling pad of a 100.00 mm thickness set at 45.00° to 45.00°, the test evaluated by Franco *et al.* [13] was then performed. To this end, a test of seven set points (in terms of frequency) in an increasing-to-decreasing sequence with non-coincident set points (Equation 6) and 100 samples per set point at a frequency of 0.33 Hz was performed in 4729.00 s. The results of similar tests with both the current and previous systems are shown in Table 4.

**Table 4:** Similar tests with the current and previous system.

	Current System	Previous System
Set points/test	12	7
Samples/set point	30	100
Sampling frequency	1.00	3.00
Time/test	1368.51	4729.00
Sampling time/test	360.00	2100.00
Settling time/test	1008.51	2629.00
Settling time/set point	84.04	375.57

#### 3.3.2. Pressure Drop Controller Evaluation

**Figure 14:**
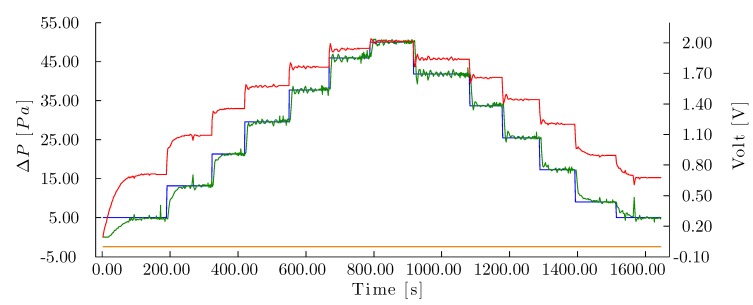
Control curve for Test 4 with the corrugated cellulose pad. (–) Pressure drop set point (Pa), (–) measured pressure drop (Pa), (–) signal to fan (V), (–) signal to pump (V).

Lastly, the pressure drop controller was evaluated using homogeneously-distributed airflow set points following the sequence described in Equation (6) without any flow in the pump (0.00 L/min). Although noise was observed in the pressure drop readings, the controller maintained the set points within the error range of ϵ=0.50 Pa (Figure 14). The test was performed in 1646.00 s, compared to 1402.00 s in Evaluation 1 for the same sequence, therefore requiring 244.00 s longer for the pressure drop controller.

### 3.4. Resulting PDAS Curves

**Figure 15:**
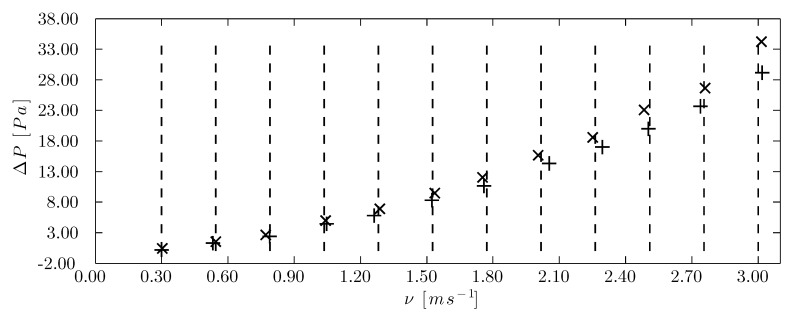
Pressure drop curve for the corrugated cellulose pad. (+) Test with constant flow of 0.0 L/min, (×) test with constant flow of 7.50 L/min and (- -) airflow speed set points (m·s^−1^).

The comparison of the pressure drop curve for the panel subjected to a constant flow compared to the same panel with no flow proved the validity of the system and the precision of the measurements. Figure 15 shows the mean pressure drop values as a function of the mean airflow speed, with the airflow set points displayed as vertical lines. Pressure drop is greater for each set point in the corrugated cellulose pad at 7.50 L/min compared to 0 L/min. In addition, there is no notable difference in the airflow speed controller precision between the system subjected to perturbation (7.50 L/min) and without it (0 L/min).

**Figure 16:**
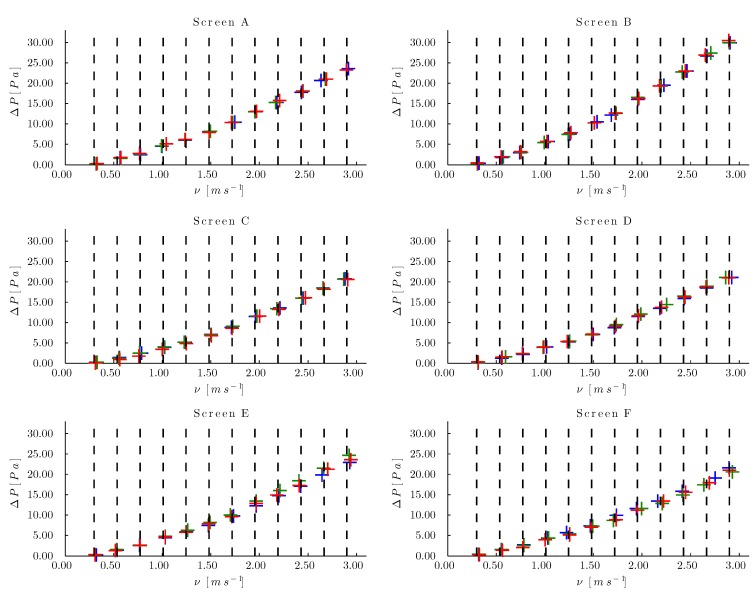
Pressure drop at the insect-proof Screens A, B, C, D, E and F. (+) Repetition 1, (+) Repetition 2, (+) Repetition 3 and (- -) airflow speed set points.

For all of the insect-proof screens, an adequate distribution of the measurement points could be observed (Figure 16) in spite of the fact that the aerodynamic characteristics of a single insect-proof screen were different for each of its repetitions, as observed clearly in mesh Ein Repetitions 1, 2 and 3, after 2.00 m/s, as indicated by the separation between the pressure drop readings. In addition, the fact that the algorithm designed a controller for each insect-proof screen improved the precision during the sampling at one set point.

### 3.5. Improvements to the Software Unit

The main improvement provided by the software unit was the implementation of the automatic PI control theory for the control of airflow speed and pressure drop in the wind tunnel, which made it possible for the software to manage the tests in terms of airflow speed and pressure drop set points, while the set points of the previous system were managed in terms of frequency.

Additionally, there was a shift compared to the previous software unit in terms of how the electrical signal information was obtained. Indeed, with the development of the driver WTD and the library ACL, communication of the software with the hardware was facilitated, resulting in a reduced need for user intervention, since it was not necessary to manage the operation of the communication port with the electronic interface.

It was estimated that the circuit improvements reduced sampling time by 66.67 % with respect to the previous software unit due to the higher sampling frequency. Likewise, the control scheme reduced settling time per set point by 77.62 % (see Table 4).

An additional improvement was the resulting flexibility when designing the tests. In the previous software unit, the algorithm to obtain the PDAS curve was developed over a set point range with the distribution of set points in terms of frequency, while in the current software unit, the same type of test was developed in terms of airflow speed or pressure drop. The newly-developed software unit featured the option of developing the test with heterogeneously-distributed set points, thus allowing a range of airflow speed values with a variety of set point densities. The increasing sequence (*Up*) expressed in Equation (3) was implemented. The capacity to assign heterogeneously-distributed set points was implemented in the algorithm, hence making it possible to generate tests with more complex sequences of changes in airflow speed or pressure drop.

Lastly, an additional substantial improvement was the ability to monitor real-time information and the mean values of the test as graphs and tables. In this way, airflow speed, pressure drop and temperature could be monitored. Moreover, the data could be directly extracted from the tables, both as averages and as recorded samples. Alternatively, the project could be saved as a MATLAB file with all of the information about the test (e.g., models, controllers, parameters of the test and test samples), and the data of the test could be directly exported to a Microsoft Excel spreadsheet. While the earlier software unit was programmed using Visual Basic, this new software unit was developed using object-oriented programming and was based on a model-view-controller architecture with the objective of facilitating future improvements to the software.

## 4. Conclusions

This paper presents a novel auto-tuning PI automatic control system for open-circuit low-speed wind tunnels for applications in greenhouse agriculture. Based on the material presented in this work, the following conclusions can be drawn:

1. The precise adjustment of specific values of airflow speed (<0.06 m·s^−1^) or pressure drop (<0.50 Pa) permitted the reproducibility and standardization of the tests. Precise mean airflow speed profiles and pressure drop values were obtained by using the feedback control technique, which resulted in only the target profiles being sent to the computer.
2. The open hardware and software platform (Arduino) has proven to be a good and cost-effective system for the developed control scheme proposed in this study. The improvements in the circuit reduced the sampling time by 66.67 % compared to the previous system, while the newly-developed control scheme reduced the settling time per set point by 77.62 %.
3. The software unit for the management of the tests developed in this paper is a research tool that facilitates the experimentation in more dynamic conditions without compromising the precision of the measurements. This is especially useful for the analysis of insect-proof screens and corrugated cellulose evaporative cooling pads. The methodology presented in this work has allowed us to substantially improve the management of the test and its flexibility. Airflow speed and pressure drop set points series could be set with a homogenous or heterogeneous distribution and be tested in different sequences. In addition, real-time monitoring made it possible to try specific set points before performing a test with the set point series.
